# First person – Paul Iyyanar and Nirpesh Adhikari

**DOI:** 10.1242/dmm.052816

**Published:** 2026-02-11

## Abstract

First Person is a series of interviews with the first authors of a selection of papers published in Disease Models & Mechanisms, helping researchers promote themselves alongside their papers. Nirpesh Adhikari and Paul Iyyanar are co-first authors on ‘ The ALX1 transcription factor acts in the early cranial mesoderm to specify extraocular muscle formation’, published in DMM. Paul conducted the research described in this article while a postdoc in Rulang Jiang's lab at Cincinnati Children's Hospital Medical Center, Cincinnati, USA. He is now a Research Scientist in the lab of Rolf Stottmann at Institute for Genomic Medicine, Nationwide Children's Hospital, Columbus, USA, investigating craniofacial genetics and developmental biology. Nirpesh is an Associate Staff Scientist in the lab of Rulang Jiang at Division of Developmental Biology, Cincinnati Children's Hospital Medical Center, Cincinnati, USA, investigating craniofacial genetics and developmental biology.


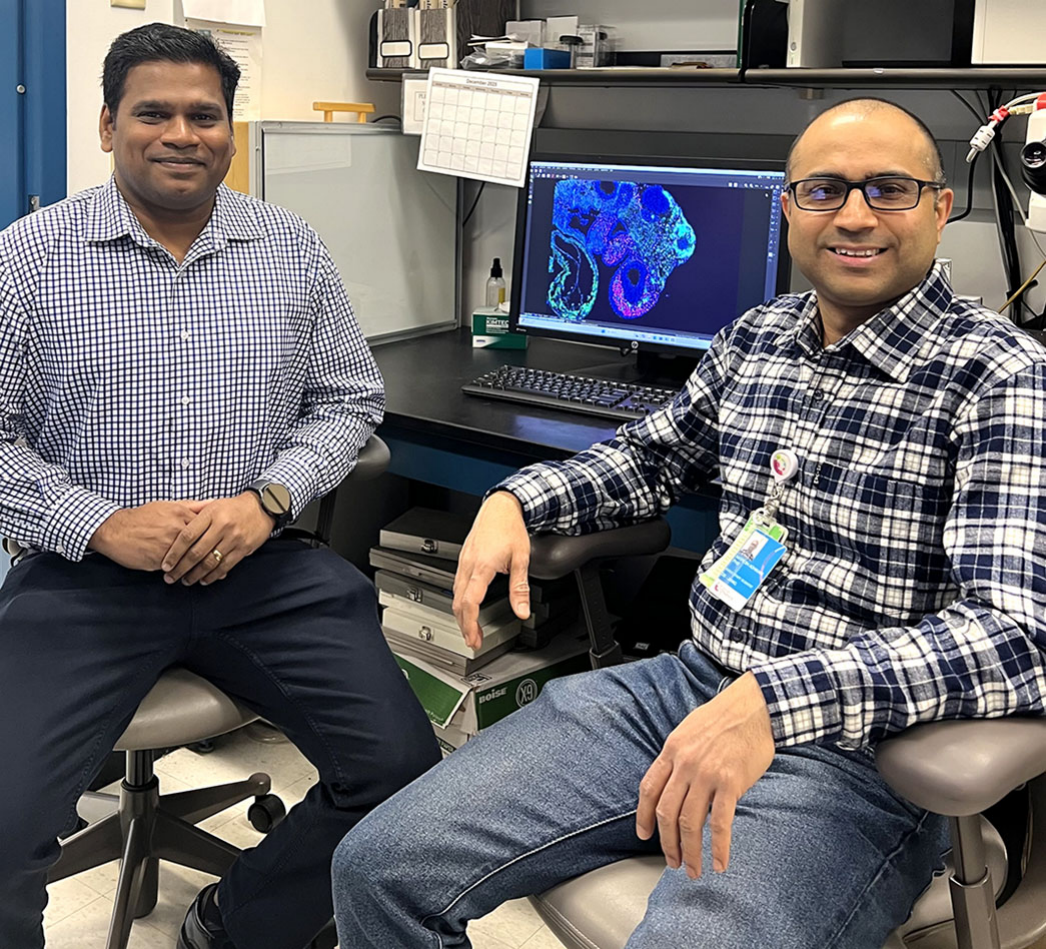


**Paul Iyyanar (left) and Nirpesh Adhikari (right)**

**Who or what inspired you to become a scientist?**

**P.I.:** My interest in science actually started in my house yard. As a teenager, I bred black and white pet rabbits, and was captivated when agouti colors unexpectedly popped up in the litters. That spark, along with guidance from two amazing mentors in high school and undergrad, set me on my research path. In my pre-doctoral work, I identified ‘what’ proteins are altered in patient samples with eye defects, but I soon realized that answering ‘how’ and ‘why’ a disease progresses requires animal models. That realization is exactly what drives my research today.

**N.A.:** During my undergrad, I was amazed to see how mouse footpad inoculation was used to culture Leprosy bacteria – the only way to really test for a cure. It hit me then that the mouse, which most people just want out of their homes, is actually an incredible laboratory model for helping humans. That realization led me to my graduate work, where I used mice to study organogenesis and mimic human diseases. I wanted to uncover the molecular mechanisms and find new therapeutic targets, and that is exactly what I am still passionate about today.

*ALX1* mutations cause severe midline facial clefts and ocular defects, including ptosis and strabismus

**What is the main question or challenge in disease biology you are addressing in this paper? How did you go about investigating your question or challenge?**

**P.I.:** *ALX1* mutations cause severe midline facial clefts and ocular defects, including ptosis and strabismus. During the initial stages of the project, we were exploring the eye defects in *Alx1* knockout mice, I fondly remember the evening I was checking the immunostaining for muscle markers and did not see any staining in the extraocular muscle (EOM) region in the *Alx1* knockout mutants. This unexpected finding of a lack of muscles that support eye movement was a major turning point for me in the project. We subsequently discovered that *Alx1* is expressed in the early mesoderm, a finding that shifted our focus.

**N.A.:** By employing tissue-specific and temporal knockout of *Alx1* gene function in mice we were able to demonstrate that ALX1 function is necessary in early cranial mesoderm for EOM development, and it is required for survival of precursor of EOM cells. Our findings have provided previously unravelled insights into the defective eye movements observed in human patients.

**How would you explain the main findings of your paper to non-scientific family and friends?**

We studied a severe condition owing to which children are born with facial clefting and eyes that do not move or open correctly. We discovered that a gene named *ALX1* acts like a ‘initiation factor’ for the muscle precursors that control the eye movement. Without this initiation factor, the early cells that are needed to build those muscles disappear before they can even start their development. By finding this missing link, we have finally explained why these eye problems happen, which helps us better understand how to support these patients.

**What are the potential implications of these results for disease biology and the possible impact on patients?**

Findings in our paper provide the first mechanistic explanation for the complex eye issues seen in *ALX1* patients. In fact, extraocular muscle defects are not even part of the official clinical diagnosis for these patients yet. For the patients and their families, this discovery is a vital step towards better clinical management. It helps doctors understand the specific developmental causes behind the eye defects, rather than just seeing the symptoms. We believe this knowledge is essential for improving the precision of diagnostic counseling and, eventually, for developing better treatments for these muscle-related ocular anomalies.

DMM felt like the most natural home for a paper that connects basic developmental biology to a real clinical challenge

**Figure:**
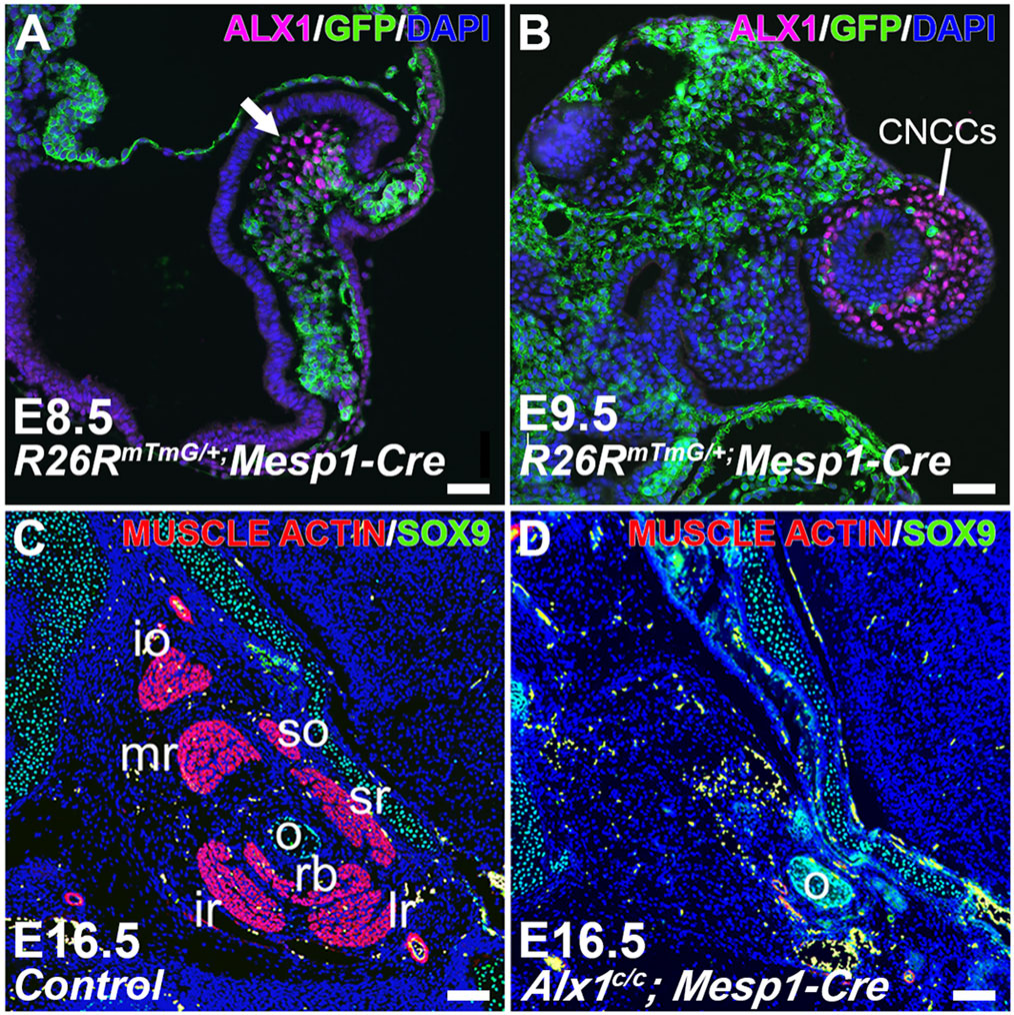
**Transient mesodermal *Alx1* expression is essential for extraocular muscle (EOM) myogenesis.** Top panels illustrate the transient window of ALX1 expression (magenta) in GFP+ cranial mesoderm at E8.5 (left), followed by its shift to neural crest lineage cells by E9.5 (right). Bottom panels demonstrate that mesoderm-specific *Alx1* inactivation leads to the total loss of all seven EOMs (right) by E16.5, compared to the organized EOMs in controls (left; muscle actin is shown in magenta).

**Why did you choose DMM for your paper?**

We chose DMM as it is a premier, highly regarded open-access journal dedicated to publishing high-quality, novel research on the mechanistic insights into human disease across diverse model systems. Since our work focuses on unraveling the mechanisms behind the ocular phenotypes in *ALX1* patients by using a novel mouse model, we felt that the journal's mission perfectly aligned with our findings. We also value that it is an open-access platform. It provides the ideal space to reach both basic scientists and clinical researchers who care about how developmental defects translate into human pathology. For us, DMM felt like the most natural home for a paper that connects basic developmental biology to a real clinical challenge.

**Given your current role, what challenges do you face and what changes could improve the professional lives of other scientists in this role?**

The rapid emergence of new technologies – particularly advancements in sequencing – presents a significant challenge for lab-based scientists, who must constantly adapt to complex analysis tools. However, this is also a unique opportunity for growth through active learning, leveraging AI platforms and fostering close collaborations with bioinformaticians. We believe that providing more-accessible, hands-on computational training for traditional wet-lab scientists would greatly improve our professional lives and bridge the gap between bench work and data analysis. In addition, it would be useful to offer sequencing data analyses as a course work in graduate studies to improve the professional lives of upcoming future scientists.

**What's next for you?**

**P.I.:** Following my postdoctoral fellowship in Rulang Jiang's lab at Cincinnati Children's Hospital, I transitioned to a Research Scientist position in the lab of Rolf Stottmann at Nationwide Children's Hospital. My immediate goal is to build upon my expertise in craniofacial and ocular development to establish an independent research career. I am eager to lead my own laboratory, where I will continue to utilize novel animal models to bridge the gap between genetic discovery and the mechanistic understanding of human disease.

**N.A.:** Having gained sufficient expertise in mouse genetics during my current postdoctoral work, I like to transition into an independent researcher in the field of craniofacial genetics and developmental biology, where I will continue to develop and utilize the animal models of human craniofacial developmental defects to understand the underlying molecular mechanism of the disease.

**Tell us something interesting about yourself that wouldn't be on your CV**

**P.I.:** Away from the lab, I like to travel, go for a swim, love meeting friends and listening to podcasts.

**N.A.:** Apart from scientific works, I love experimenting with food in the kitchen and adding different flavors in foods. Playing with mechanical tools for creating shapes and fixing things in the garage are some of things I routinely spend time on. I also value helping people in need and doing charity work.

## References

[DMM052816C1] Iyyanar, P. P. R., Adhikari, N., Lan, Y. and Jiang, R. (2026). The ALX1 transcription factor acts in the early cranial mesoderm to specify extraocular muscle formation. *Dis. Model. Mech.* 19, dmm052241. 10.1242/dmm.052241 41670220 PMC12937918

